# Dissecting the Functional Role of the TRIM8 Protein on Cancer Pathogenesis

**DOI:** 10.3390/cancers14092309

**Published:** 2022-05-06

**Authors:** Jessica Elisabetta Esposito, Vincenzo De Iuliis, Francesco Avolio, Eliana Liberatoscioli, Riccardo Pulcini, Simona Di Francesco, Alfonso Pennelli, Stefano Martinotti, Elena Toniato

**Affiliations:** 1 Center of Advanced Studies and Technology, Department of Innovative Technology in Medicine and Dentistry, University of Chieti, 66100 Chieti, Italy; j.elisabetta.esposito@gmail.com (J.E.E.); avolio.francesco@gmail.com (F.A.); elianaliberato.scioli@gmail.com (E.L.); riccardo.pulcini@unich.it (R.P.); smartinotti@unich.it (S.M.); 2 Department of Clinical Pathology, G. Mazzini Civil Hospital, ASL 4, 64100 Teramo, Italy; vincenzo.deiuliis@aslteramo.it; 3 Department of Medical, Oral Sciences and Biotechnology, University of Chieti, 66100 Chieti, Italy; docveronica@gmail.com (S.D.F.); a.pennelli@dsb.unich.it (A.P.); 4 Department of General Pathology, UniCamillus-International Medical University in Rome, 00100 Rome, Italy

**Keywords:** TRIM proteins, TRIM8, p53, NF-kB, JAK-STAT, cancer

## Abstract

**Simple Summary:**

The tripartite motif (TRIM) gene family is a large group of E3 ubiquitin ligase proteins that can also have proteasome-independent functions. This review summarizes the structural organization, the biological functions and the mechanisms involved in cancer pathogenesis of TRIM proteins. Furthermore, this paper focuses on TRIM8, a member of the TRIM family proteins, describing its role both as a tumor suppressor and as an oncogene.

**Abstract:**

TRIM/RBCC are a large family of proteins that include more than 80 proteins, most of which act as E3 ligases and catalyze the direct transfer of Ubiquitin, SUMO and ISG15 on specific protein substrates. They are involved in oncogenesis processes and in cellular immunity. On this topic, we focus on TRIM8 and its multiple roles in tumor pathologies. TRIM8 inhibits breast cancer proliferation through the regulation of estrogen signaling. TRIM8 downregulation in glioma is involved in cell proliferation, and it is related to patients’ survival. Several studies suggested that TRIM8 regulates the p53 suppressor signaling pathway: it is involved in the NF-kB pathway (Nuclear Factor kappa light- chain-enhancer of activated B cells) and in STAT3 (Signal Transducer and Activator of Transcription 3) of the JAK-STAT pathway. In this review, we summarize how the association between these different pathways reflects a dual role of TRIM8 in cancer as an oncogene or a tumor suppressor gene.

## 1. Introduction

TRIM8 is a part of a huge of proteins known as Tripartite Motif proteins, in the first instance described as a glioblastoma expressed RING-finger protein (GERP) [1]. They are also known as the RING family and are a large family of proteins characterized by the presence of three characteristic domains in the high-conserved N-terminal region: RING domain, B-box domain and coiled-coil region [2,3,4].

**Figure 1:**
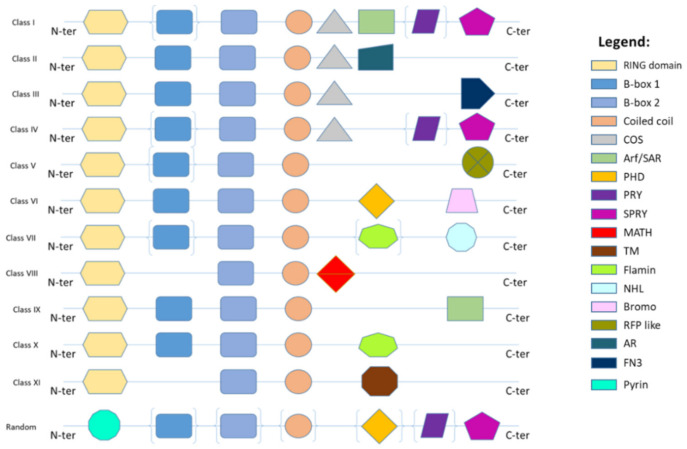
Classification of TRIM/RBCC proteins. The N-terminal domain (N-ter) is mostly conserved in TRIM family members and includes RING, B-box 1, B-box 2 and coiled-coil domains. A variable C-terminal domain (C-ter) classifies TRIMs into 12 different classes and includes COS box motif, ARF (ADP ribosylation factor)/SAR, PHD (Plant Homeodomain), PRY, SPRY (SPla and the RYanodine receptor), MATH (meprin and TRAF homology domain), TM (transmembrane motif), FILAMIN, NHL (NCL1/HT2A/LIN-41), Bromo domain, FN3 (FibroNectin type III motif), and a variable domain. The presence of certain domains can vary even among members of the same class, as indicated by brackets.

TRIM proteins are ontogenetically preserved [5,6]. Their primary sequences demonstrate a relatively low analogy, except for a few members. In fact, exclusively the cysteine and histidine characterizing the RING and B-box domains and the hydrophobic residues of the coiled-coil region are highly preserved given that they are mandatory to sustain the scaffold structure of the proteins [7,8]. TRIM proteins are able to integrate into high molecular weight complexes through the association of coiled-coil domains. These complexes are located in particular sub-compartments, such as cytoplasmic bodies or ribbon-like structures, which can be allocated around the nucleus (e.g., TRIM13) or in the nucleus where they constitute “nuclear bodies” (e.g., TRIM8, 19, 30 and 32) or “nuclear sticks” (e.g., TRIM6). Few members (e.g., TRIM24, 28 and 33) contain the bromo domain, which in the nucleus interacts with the acetylated lysines of histones. Only some members do not have a RING domain but are still considered TRIM/RBCC proteins because they conserve all the other domains (B-boxes and coiled-coil region) in the identical sequence as the other members [9,10,11]. The C-terminal region is less conserved than N-terminal and could present different protein-protein association domains, on the basis of which they are categorized in 11 sub-groups [2,3,4,12] (Figure 1).

On this topic, we firstly described TRIM proteins’ biological functions and the mechanisms and pathways implicated in cancer pathogenesis. Later, we focused on TRIM8 protein and its dual role both as an oncogene by affecting the NF-kB and JAK-STAT pathways and as a tumor suppressor by inducing TP53-dependent cell cycle arrest.

## 2. TRIM Proteins Biological Functions

TRIM proteins are involved in distinct cellular processes, despite showing a similar structure: regulation of cellular homeostasis, cell cycle, senescence, apoptosis, differentiation, specific metabolic pathways, meiosis and protein quality control [9,13,14]. They exert their actions on transcriptional regulation, cytoskeletal remodeling, intracellular trafficking, membrane repair and oncogenesis [15,16,17,18]. Moreover, these proteins are implicated in the development and regulation of the immune system [19,20,21].

Most TRIM molecules act as E3 ligases and directly catalyze the transfer of Ub, SUMO and ISG15 on specific protein substrates [22,23,24,25]. The conjugation reaction of ubiquitin to a substrate is catalyzed by E1 ubiquitin-activating enzyme, E2 ubiquitin-conjugating enzymes and E3 ubiquitin ligases [26]. The E3 ubiquitin ligases can be divided into two major classes: the homologous to E6-AP COOH terminus (HECT) E3 ubiquitin ligase family and the RING-finger-containing E3 ubiquitin ligase family [27,28]. The high number of E3 ligases is associated with their specificity in selectively targeting protein substrates [9]. Mostly, the enzymatic activity exerted by the E3 ligases on ubiquitin and Ubiquitin-like molecules (UBL) depends on the presence in the protein structure of the RING domain [29,30]. E3 ligases transfer the ubiquitin or UBLs from E2 conjugating enzymes to the substrates, and thus they are responsible for recognizing the substrates and are determinants of target specificity [31]. In order for ubiquitination to take place, however, it is not sufficient for the RING domain to recognize the specific substrate, but specific functional protein dimers must be formed [32,33,34]. The B-box and coiled-coil domains are responsible for the dimerization of TRIM proteins [2,35].

Many TRIM proteins play a pivotal role during mitosis and cell-cycle progression. Specifically, TRIM19, TRIM22, TRIM28, TRIM37 and TRIM6 are important during prophase; TRIM19, TRIM32 and TRIM69 in prometaphase; TRIM17, TRIM36 and TRIM69 in metaphase; and TRIM17, TRIM21, TRIM47 and TRIM76 in cytokinesis. Furthermore, in the course of the bipolar spindle assembly during all phases of mitosis, TRIM8 is involved in the mitotic spindle formation through interaction with two important regulators of mitotic spindle machinery and cytoskeleton reorganization, KIF11 and KIFC1, and through localization at the mitotic spindle [36]. TRIM8 colocalizes on centrosomes with Plk1 and straight reacts to CEP170-like protein. This interaction, suppressing TRIM8 function, induces a delay of the mitosis progression with a cell accumulation in the G2/M phase. TRIM8 is also necessary for chromosomal stability. As a matter of fact, the suppressing of TRIM8 induced an increased rate of chromosomal instability leading to a significant rise of cells with less than 46 chromosomes [9,27].

TRIM proteins have the distinctive feature of exerting a large variety of different roles and activities because of their ubiquitination or ubiquitin-like function that labels the target proteins to be degraded at the proteasome level, as well as stabilize or dislocate them in various cellular compartments through such modifications. Ubiquitination is a post-transductional modification of protein substrates necessary for different biological mechanisms, such as:

- Regulation of the activity and stability of oncogenes and tumor suppressors [37,38];
- Degradation of toxic protein aggregates [39];
- Activation of specific inflammatory pathways [15,40].

The alteration of the post-transduction mechanism of ubiquitination affects the functionality of protein substrates, with consequent alteration of the biological mechanisms in which they are involved. At a macroscopic level, these alterations can lead to the development of various pathological conditions, including tumor pathologies [2,41,42]. TRIM proteins are involved in carcinogenesis. In particular, these proteins are implicated in several biological functions: DNA repair, metastasis, tumor-suppressive and oncogenic regulation [15]. Furthermore, some of the TRIM family proteins play a pivotal role in autophagy and innate immunity and regulate important cellular processes, such as intracellular signaling and transcription [27]. The down-regulation or overexpression of TRIM proteins has long been investigated in the study of oncogenesis. However, many reports showed that TRIM alterations were observed in lung cancer, breast cancer, liver cancer, colorectal cancer and prostate cancer [43,44]. Indeed, reduced expression of these proteins could reflect the suppressive role of the tumor, whereas their over-expression could reflect their contribution to the disease development and/or progression. Therefore, some TRIMs could be considered biomarkers for some kind of cancer. In particular, TRIM11, TRIM14, TRIM24, TRIM25, TRIM27, TIM28, TRIM29, TRIM33, TRIM37, TRIM44 and TRIM59 are the most associated with cancer [43].

## 3. TRIM Proteins and Cancer Pathogenesis

TRIM proteins could influence cancer pathogenesis through the following mechanisms:

- Chromosomal translocation [45]. It could generate a fusion protein without activity or with a different activity that could dysregulate some signaling pathways, leading to the generation of some tumor shapes. An example is a translocation between the TRIM19 gene (PML) on chromosome 15 and the retinoic acid receptor α (RARa) gene on chromosome 17. This translocation leads to the formation of a fusion protein that represses acid signaling retinoic and is associated with Acute Promyelocytic Leukemia [46]. Such similar examples are the following: TRIM24, TRIM27 and TRIM33 were found in translocations with the RET gene and are involved in papillary thyroid cancer, lymphoma and non-small cell lung carcinoma, respectively. Similarly, TRIM24 was found translocated with the BRAF gene in melanoma and lung cancer and with the FGFR1 gene in myeloproliferative syndrome [43];
- Modulation of the activity and stability of p53. TRIM11, TRIM13, TRIM21, TRIM24, TRIM25, TRIM28, TRIM29, TRIM31, TRIM32, TRIM39 and TRIM59 can ubiquitinate the p53 protein, a fundamental macromolecule in cell development whose purpose is to promote genomic stability and induce cell cycle arrest and apoptosis if extensive DNA damage is found in the cell. The ubiquitination of this protein leads to its direct degradation or to its sequestration in the cytoplasm: since it can no longer penetrate the nucleus, the ubiquitinated protein is no longer able to detect any damage to the DNA; consequently, the cell replicates itself by transmitting the same error in the nucleic acid sequence, resulting in the possible onset of tumor forms [47];
- Regulation of pathways to cancer stemness, including STAT signaling, AKT signaling, NANOGSox2-Oct-3/4 networks. Specifically, through these pathways, TRIM28 is involved in breast cancer, TRIM24 in glioblastoma and colorectal cancer, TRIMs 14 in gastric cancer and TRIM16 has been associated as a negative regulator of stemness in breast and ovarian cancer cells [43].

**Figure 2:**
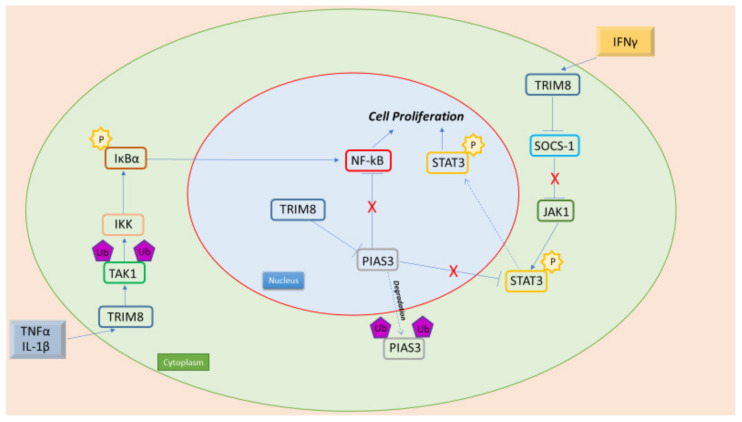
TRIM8 is an oncogenic protein, with its interaction with NF-kB and STAT3 leading to cell proliferation. Pro-inflammatory cytokines (TNFα e IL-1β) promote NF-kB activation through TRIM8. In fact, TRIM8 promotes TAK1 Lys63- linked polyubiquitination, leading to IKK kinase activation. Moreover, in the nucleus, TRIM8 promotes the translocation of PIAS3 in the cytoplasm, where it is then degraded. PIAS3 in the nucleus interacts with NF-kB preventing its activation. Furthermore, TRIM8 induces the activation of the JAK-STAT pathway promoted by IFN-γ through the degradation of two STAT protein inhibitors, PIAS3 and SOCS-1.

Stem cells (SCs) are cells with no signs of differentiation, capable of self-renewing and generating progeny capable of differentiating into different cell types. They constitute the reserve elements of human tissues; in fact, they are activated only to restore tissue damage or to ensure normal cell turnover. SCs are capable of self-renewal, are multi-potent and immortal, and are highly resistant to chemical and physical agents, all characteristics also possessed by cancer cells. Furthermore, SCs tend to maintain the ability to de-differentiate in order to return to a primitive state of development. Such cells cannot survive outside their environment or in case of deficiency of specific cytokines and growth factors. Mutated stem cells, however, despite having all the aspects of stem cells, are unable to support tissue homeostasis, favoring, instead, the onset and progression of tumor diseases. The stem characteristics common to HF and cancer cells provide the building blocks for cancer maintenance and survival, from the potential for self-renewal and differentiation to the organization of microenvironments that support stemness. Thus, cancer stem cells (CSCs) are defined as the small population of cells within tumors that possess stem properties that support cancer development, such as advanced capabilities for cloning, growth, metastasis, re-proliferation and self-renewal. CSCs exhibit remarkable organizational skills. In fact, they can educate neighboring cells to provide nutrients and collaborate in evading the immune system, thus creating an environment favorable for tumor progression [48]. CSCs give rise to heterogeneous cell populations, often with a high potential for plasticity, high resistance to stressors, such as low oxygen or nutrient levels, or the initiation of cell death by chemotherapeutic agents, and the capability for quiescence as a typical response to these factors [49]. Among the possible pathways by which TRIM proteins act on tumor stemness are the signaling of STAT, AKT (Figure 2) and the NANOGSox2-Oct-3/4 networks [50]. In fact, TRIM proteins control stem cell characteristics mostly positively by enhancing the activity of core transcription factors, induction of specific signaling pathways, epigenetic silencing of pro-differentiation genes, metabolic reprogramming and activation of the epithelium-mesenchymal transition pathway. Several members, such as TRIM24 and TRIM28, negatively regulate stem cell self-renewal, presumably by ubiquitin-mediated degradation of stem cell transcription factors or inhibition of specific signaling pathways. Furthermore, several TRIM proteins, such as TRIM8, modulate the stem cell phenotype both positively and negatively [48]. In addition, the role of TRIM proteins on autophagic processes may represent a not-well investigated mechanism involved in cancer stemness [43].

- Regulation of pathways related to epithelial-mesenchymal transition (EMT) [43,51].

The epithelium-mesenchymal transition (EMT) is a biological process that allows a polarized epithelial cell, which normally reacts with the basement membrane through its basal surface, to suffer several biochemical changes that permit it to acquire a mesenchymal cell phenotype, which includes a prominent migratory ability, invasiveness, significant opposition to apoptosis and considerably raised production of components of the extracellular matrix. The achievement of an EMT is signaled by the degradation of the underlying basement membrane and the development of a mesenchymal cell that can move away from the epithelial layer where it arises [52].

The biological processes that initiate an EMT include activation of specific transcription factors, expression of cell surface proteins, reorganization and expression of cytoskeletal proteins, production of degradative enzymes and changes in the expression of specific microRNAs. Some of these factors can be used as biomarkers to establish the switch of a cell through an EMT [53].

## 4. TRIM8

TRIM8 gene (Ensembl: ENSG00000171206) is situated on chromosome 10q24.32, a region that is known to show frequent deletion or loss of heterozygosity in glioblastomas [1]. Fifteen splice-variant transcripts of TRIM8 were recognized in humans. Surprising enough, three of them are uncharacterized long non-coding RNAs (lncRNAs) (Ensembl). It has been shown that TRIM8 is present in 27 human tissues considered in the Human Protein Atlas (HPA) RNA-Seq Project and produces one major transcript (Ensembl: ENST00000643721.1) of 7290-bp length that codes for the 551-amino acid (aa) TRIM8 protein with a molecular mass of 61.489 kDa (UniProtKB: Q9BZR9 TRIM8 HUMAN) [27].

The human TRIM8 is an E3 ubiquitin ligase protein and is composed of an N-terminal C3HC4-type RING-finger domain, two B-boxes (Bbox1 and Bbox2), a coiled-coil domain and a proline-rich no-domain C-terminal region with a monopartite nuclear localization signal (NLS) (Figure 1) [54]. TRIM8 is structurally classifiable under class V of TRIM-type proteins, along with TRIM19, TRIM31, TRIM40, TRIM56, TRIM73, TRIM74, RNF207, TRIM52 and TRIM61, which are also characterized by not having known domains in their C-terminal region so far [43]. The RING domain of TRIM8 plays an important role in its activity to regulate stabilization and activation of TP53, degradation of MDM2,8 and destabilization of DNp63a,9 and thus is crucial in cell proliferation. Instead, it was shown that the conserved B-box and coiled-coil domain region is relevant to mediating the interaction with SOCS1, a tumor suppressor gene. Moreover, the coiled-coil domain of TRIM8 is necessary for homodimerization and allows the formation of Nuclear Bodies (NBs) in order to regulate the activity of main cellular proteins through protein–protein interactions. Deletion of the C-terminal domain of TRIM8 can result in protein mislocalization [9,27].

TRIM8, as a whole, is considered to be among E3 ubiquitin ligases due to the presence of the RING domain. TRIM8 was shown to function as an E3 ubiquitin ligase in various relevant biological pathways, although the mechanism of its E3 ubiquitin ligase activation is not known yet. TRIM8 can carry out K63-, K6- and K33-linked polyubiquitination [55]. Specifically, TRIM8 can react with Toll/IL-1 receptor domain-containing adaptor-inducing IFN-b (TRIF) and regulates its K6- and K33-linked polyubiquitination, which promotes the disruption of the TRIF-TANK-binding kinase-1 association [56]. In addition, TRIM8 acts as an important regulator of TNF-a- and IL-1b-induced NF-kB activation through the K63-linked polyubiquitination of TGF-b-activated kinase 1 (TAK1), mediated by TNF-a and IL-1b [57]. K63-linked ubiquitination is required in regulating proteasome-independent functions, including cellular processes, such as endocytosis and inflammatory immune responses, innate immunity, protein trafficking and NF-kB signaling. Instead, K6-linked polyubiquitination is known to be related to DNA damage response and Parkin-mediated mitophagy, and K33-linked polyubiquitination is associated with TCR signaling, post-Golgi-trafficking and AMPK-related kinase signaling. Recent studies demonstrated that TRIM8 is not only involved in an E3 ubiquitin ligase-independent manner, but it can also preserve phosphorylated IRF7 (pIRF7) from proteasomal degradation through an E3 ubiquitin ligase-independent pathway by avoiding its recognition by the peptidylprolyl isomerase [27].

## 5. TRIM8 and Cancer Pathogenesis

TRIM8 regulates the tumor suppressor p53 [58], the NF-kB [55] (Nuclear Factor kappa light- chain-enhancer of activated B cells) and the STAT3 [59] (Signal Transducer and Activator of Transcription 3) of the JAK-STAT pathways; its association with these three pathways explains its dual role in cancer as an oncogene or as tumor suppressor protein [9]. TRIM8 was demonstrated in murine and human tissues with greater expression in the central nervous tissue, kidney and lens; it was also proved in undifferentiated embryonic stem cells, indicating that TRIM8 could play a relevant role in maintaining pluripotency.

TRIM8 is involved in most tumors as a suppressor gene. In fact, its downregulation was observed in glioblastoma, clear cell renal cell carcinoma (ccRCC), larynx squamous cell carcinoma (LSCC), colorectal cancer (CRC), chronic lymphocytic leukemia (CLL), osteosarcoma cell lines and breast cancer (BC) [27].

Firstly, the role of the TRIM8 gene in tumors was described by Vincent et al. in 2000 [1]. They observed that patients affected by glioblastomas were characterized by a frequent deletion or loss of heterozygosity in the TRIM8 gene, which causes the loss of gene copy number associated with the inactivation of TRIM8 in glioblastoma cells. Indeed, deficiency in TRIM8 E3 ligase function in glioma cells might stimulate cancer development by promoting the oncogenes stabilization and/or increasing tumor suppressors degradation. Compared with cells at highest TRIM8 levels, lowest TRIM8 expression levels related to a relevant risk of death and disease development in WHO grade III tumors, indicating that a loss of TRIM8 expression may be required for the transformation to a more aggressive phenotype typical of WHO grade III gliomas. Moreover, it was shown both U87MG glioblastoma and patients’ primary glioma cell lines were characterized by the overexpression of TRIM8, which suppresses cell development and induces an important decrease in clonogenicity [60].

Moreover, in breast cancer, downregulated TRIM8 was associated with a poor prognosis. In particular, Tian et al. showed a TRIM8 downregulation in breast cancer and an inverse correlation between the protein level of TRIM8 and the estrogen receptor α (Erα). In ER-positive BC, TRIM8 reacts with the AF1 domain of estrogen receptor α through its RING domain in the cytoplasm and rises poly-ubiquitination of the ERα protein, causing degradation of ERα. TRIM8 binds ERα protein and catalyzes its K48-linked ubiquitination to block K63-linked ubiquitination, thus promoting ERα degradation, which further inhibits K63-linked poly-ubiquitination. TRIM8 may be a potential therapeutic target in the ER-positive BC treatment, as evidenced by the post-translational mechanism between ERα and TRIM8 [61].

In several cases, the expression of TRIM8 is modulated at transcriptional and post-transcriptional levels by microRNAs (miRNAs). For instance, the overexpression of miR-17-5p demonstrated in patients affected by ccRCC, CRC, Glioma and CLL results in TRIM8 downregulation that affects cell proliferation and is related to patients’ survival [62]. Micale et al. suggested that TRIM8 and miR-17 were involved in a feedback pathway implicated in glioma pathogenesis. The authors evidenced that miR-17 plays a pivotal role in glioma cell lines: the downregulation of this miRNA significantly decreases cell viability and raises apoptotic function, while the upregulation is related to advanced tumor development and poor overall survival [60].

In cell Renal Cell Carcinoma miR-17-5p, miR-106b-5p and miR-182 bind TRIM8 mRNA leading to its degradation [9]. In colorectal cancer and in anaplastic thyroid cancer (ATC), TRIM8 seems to be associated with chemoresistance of CRC and ATC cells, respectively. In particular, in CRC, TRIM8 downregulation was promoted by the overexpression of miR-17-5p and miR-106b-3p induced by N-MYC. In addition, N-MYC is negatively regulated by miR-34a, which is transactivated by p53. Silencing of miR-17-5p and miR-106b-5p leads to a raise of TRIM8 expression levels, which in turn conducts to p53 stabilization with the activation of cell cycle arrest and transcription of miR-34a that targets N-MYC for degradation. Thus, the oncogenic effect of N-MYC is suppressed by p53 through the transcription of miR-34a, linking p53 to N-MYC. TRIM8 acts as a key point in the p53/N-MYC/miR-17 pathway. CRC cells recover sensitivity to chemotherapy treatments by restoring normal TRIM8 expression levels. Finally, in anaplastic thyroid cancer (ATC), TRIM8 is a direct target of miR-182, which is upregulated in ATC tissue and cell lines. Silencing the function of TRIM8, miR-182 promotes cellular development and increases the chemoresistance of ATC cells [62].

## 6. TRIM8 as Tumor Suppressor

Several studies reported that TRIM8 has a key role both as an oncogene by affecting the NF-kB [55] and JAK-STAT pathways [59] and as a tumor suppressor [62]. In particular, a positive feedback loop involving TRIM8 and p53 was described. This pathway is activated in response to genotoxic stress causing p53 stabilization and activation, resulting in cell cycle arrest and decreasing in cell proliferation. TRIM8 physically reacts with p53, avoiding its association and degradation by the principal negative regulator of p53, Murine Double Minute 2 (MDM2) [9]. TRIM8 replaces p53-MDM2 binding, thus stabilizing p53 and stimulating MDM2 degradation. Finally, TRIM8 act as a promoter of cell proliferation and DNA repair through p53-dependent suppression [62]. The combined activation of TRIM8 and p53 can result in negative outcomes in response to hypoxic stress due to ischemia following a stroke or myocardial infarction.

TRIM8 deficiency avoided p53 activation following drug treatments in several p53 wild-type cell lines. For example, in RCC showing wild-type p53, the loss of one copy of the gene causing downregulation of TRIM8 was shown to impair the p53-mediated cellular responses to chemotherapeutic drugs in renal cell carcinoma. TRIM8 expression recovery in RCC cell lines makes these cells sensitive to chemotherapeutic treatments following p53 pathway reactivation. As a consequence, TRIM8 could be used as a factor that improves the chemotherapy efficacy in cancers where p53 is wild-type, and its pathway is defective. Moreover, reactivation of the p53 pathway can be confined exclusively to cancer cells without affecting normal tissue, hence limiting side effects. One mechanism to reactivate p53 in tumor types harboring a wild-type p53 is to constrain the maintenance of p53 protein by releasing it from the negative control of MDM2 [63].

The TRIM8 ability to prevent the proliferation of cancer cells is also related to its effects on the stability and activity of the oncogenic transcription factor DNp63, which is included in the p53 gene family. As it is upregulated in many different tumors, DNp63 expression is associated with a poor prognosis [64]. DNp63 degradation is carried out by TRIM8 in both proteasomal and caspase-1 dependent ways, but DNp63 is able to downregulate TRIM8 transcription expression levels, thus avoiding p53 stabilization [9].

TRIM8 can perform as a tumor suppressor by inducing TP53-dependent cell cycle arrest [64]. In summary, the TRIM8 anticancer capacity has expertise in three distinct ways, in all of which TRIM8′s anti-proliferative function is affected by the TP53 functional or wild-type background:

- By inducing the TP53 tumor suppressor activity through a positive feedback loop formation.

TRIM8, a direct target gene of TP53, promotes TP53 expression and tumor suppressor activity through a positive feedback loop-forming mechanism with TP53 during UV-instigated stress factors by triggering cell cycle arrest genes such as CDKN1A (p21) and GADD45 expression. The recovery of TRIM8 expression, downregulated in many tumors, can lead to the enhancement of efficacy of chemotherapeutic drugs by reactivating the TP53 pathway. Notably, TRIM8 silencing prevents TP53 activation after UV radiation [64];

- Restoring TP53 functions by blunting N-MYC activity in chemo-resistant tumors.

The inhibition of miR-17-5p and/or miR-106-5p leads to the restoration of TRIM8-mediated TP53 tumor suppressor activity and inhibits N-MYC-dependent cell proliferation through miR-34a upregulation [65];

- Quenching the DNp63a oncogenic activity by forming a negative feedback loop.

TRIM8 can blunt the pro-proliferative function of oncogenic DNp63a in a TP53 wild-type background [27]. The transcription factor ∆Np63α is upregulated in several tumors, and its expression level is associated with a poor prognosis. TRIM8 affected the degradation of ∆Np63α in both a proteasomal and caspase-1-dependent expertise pathway, opposing the proliferation of cancer cells [64].

## 7. TRIM 8 as Oncogenic Protein

TRIM8 has been shown to play a role both as an oncogene and as a tumor suppressor, thus allowing the proliferation of cancer cells. TRIM8 enables autophagic processes mediating lysosomal biosynthesis and autophagy flux in a p53-independent manner. Autophagy preserves cellular homeostasis by removing deteriorated proteins, aggregates and defective organelles and eliminating damaged DNA, which, following genotoxic stress, is transported out of the nucleus and degraded by the lysosomes. TRIM8 regulates the expression of p62, which is involved in several functions during autophagic processes such as being a cargo selector, inflammation and senescence induced by DNA damage and decreasing inflammation by promoting mitophagy. In fact, during genotoxic stress, TRIM8 can allow a proliferative advantage to cancer cells by increasing autophagy flux through lysosomal biogenesis and inactivating the cleaved Caspase-3 subunit to inhibit cell death induced by genotoxic stress. TRIM8 knockdown reduces the expression of X-linked inhibitor of apoptosis protein (XIAP), a major regulator of cell death and autophagy, whereas the enhanced expression of TRIM8 stabilizes XIAP, forming a trimeric complex with Caspase-3, inhibiting XIAP activation in the presence of etoposide. XIAP also strongly activates NF-kB via BIR (baculovirus inhibitor of apoptosis protein repeat) domain-mediated dimerization and binding to TGF-b-activated kinase 1 (MAP3K7) binding protein. This XIAP-mediated NF-kB activation also induces the expression of genes involved in autophagy, such as Beclin-1 [9,27,66].

During genotoxic stress, TRIM8-mediated XIAP stabilization can also promote the degradation of Caspase-3, one of the most important players in the apoptotic cascade. For that reason, TRIM8-mediated XIAP stabilization has the capability to lead to two important oncogenic results during the course of tumorigenesis. First, TRIM8-mediated XIAP stabilization allows the expression of genes involved in autophagy and cell proliferation through NF-kB activation. Second, TRIM8 mediated stabilized XIAP averts activation of Caspase-3, leading to the suppression of apoptosis. Therefore, TRIM8 avoids cell death during genotoxic stress and radiation therapy, suggesting that TRIM8′s highly oncogenic potential can allow survival assistance to cancer cells [27].

### 7.1. TRIM8 and NF-kB

The principal TRIM8 partners involved in its role in oncogenesis are NF-kB and STAT3. NF-kB consists of a family of inducible transcription factors that control the expression of numerous genes that play a pivotal role both in immune and inflammatory responses. The classical activation of NF-kB is initiated by the phosphorylation of the NF-kB inhibitor IkBα through the activated IkBα kinase complex (IKK). Phosphorylation of IkBα is followed by its consequent ubiquitin proteasomal degradation, which promotes the release of the NF-kB dimers with subsequent nuclear entry. Once inside the nucleus, NF-kB dimers modulate genes implicated in cell death inhibition and cell proliferation, thus stimulating migratory and invasive phenotypes associated with tumor progression as well as Epithelial–Mesenchymal Transition (EMT) [52]. TRIM8 can activate the NF-kB signaling pathway both in the cytoplasm and in the nucleus. In the nucleus, the translocation of PIAS3 (Protein Inhibitor of Activated STAT3) from the nucleus to the cytosol performed by TRIM8 induces PIAS degradation. In this way, PIAS3 can no longer bind the RelA (p65) subunit of NF-kB, which is free to dimerize and activate the NF-kB responsive genes. In the cytoplasm, TRIM8 enhances the activation of NF-kB triggered by TNFα, the most important activator of carcinogenesis and inflammatory diseases, and IL-1β. TRIM8 regulates the polyubiquitination of TAK1, which in turn activates the kinase IKK, which promotes IkBα phosphorylation and NF-kB activation (Figure 2).

TRIM8 plays a relevant role in regulating tumor necrosis factor-alpha (TNF-a) and interleukin (IL)-1b-induced nuclear-factor kB (NF-kB) activation through the K63-linked polyubiquitination of TAK1. In fact, overexpression of TRIM8 activates NF-kB and enhances TNF-a- and IL-1b-induced activation of NF-kB, whereas knockdown of TRIM8 leads to inverse effects. TRIM8 also mediates the proliferation and migration ability of the cells through the NF-kB pathway, and the knockdown of TRIM8 in the breast cancer MCF7 cell line significantly reduces the cell proliferation and clonogenicity of cells (Figure 2) [57].

The activation of NF-kB is responsible for cell proliferation and protecting cells from initiating apoptosis. Thus, TRIM8 plays an important role as an oncogene involved in cell proliferation by positively mediating the TNF-induced NF-kB pathway [27].

### 7.2. TRIM8 and STAT3

STAT3 is a transcriptional factor belonging to the STAT family of transduction signal responsive transcription factors. Similar to NF-kB, STAT3 is also retained in an inactive form in the cytoplasm of non-stimulated cells. The phosphorylation of Tyr 705 of STAT3 is necessary for its dimerization and, thus, activation. In the dimerized form, STAT3 is able to enter the nucleus and promotes the transcription of several target genes. Members of the JAK family of tyrosine kinase receptors commonly mediate STAT activation, and in the case of STAT3, the major activator is JAK1. Moreover, STAT3 activity can be optimized through a reversible acetylation mechanism, which also influences the activity of NF-KB family members [67]. TRIM8 association with SOCS-1 through the SH2 domain promotes its degradation and allows the activation of JAK-STAT induced by IFNγ (Figure 2) [17].

Both NF-kB and STAT3 are activated in response to overlapping stimuli, such as stresses and cytokines, although they are regulated by entirely different signaling mechanisms. Once activated, both NF-kB and STAT3 regulate the expression of several genes involved in pro-proliferative pathways, immune response and anti-apoptotic processes [9].

## 8. Conclusions

TRIM/RBCC is a large family of proteins, most of which act as E3 ligases, involved in cellular signaling, metabolism, autophagy, oncogenesis processes and in cellular immunity. The alteration of the post-transduction mechanism of ubiquitination affects the functionality of protein substrates, with consequent alteration of the biological mechanisms in which they are involved. These alterations can lead to the development of various pathological conditions, including tumor pathologies. In fact, TRIM proteins play a critical role in carcinogenesis and are involved in several biological processes, such as DNA repair, metastasis, tumor-suppressive regulation and oncogenic regulation. Furthermore, some of the TRIM family proteins are relevant factors required for autophagy and innate immunity and regulate significant cellular processes, such as intracellular signaling and transcription. The down-regulation or overexpression of TRIM proteins was described in lung cancer, breast cancer, liver cancer, colorectal cancer and prostate cancer. Indeed, reduced expression of these proteins could reflect the suppressive role of the tumor, whereas their over-expression could reflect their contribution to the disease development and/or progression.

In this review, we focused on TRIM8 and its multiple roles in tumor pathologies. Specifically, TRIM8 regulates the p53 suppressor signaling pathway; it is involved in the NF-kB and in STAT3 of the JAK-STAT pathways. In this review, we also summarized how the association between these different pathways reflects a dual role of TRIM8 in cancer as an oncogene or tumor suppressor gene. However, many experiments enhance the anti-oncogenic function of the protein. In particular, it was shown that TRIM8 physically interacting with the oncosuppressor p53 protein increases its stability leading to cell cycle arrest. For these reasons, TRIM8 could be indicated as a potential target capable of enhancing the p53-mediated tumor suppressor activity.
